# Clinical observations on chemotherapy curable malignancies: unique genetic events, frozen development and enduring apoptotic potential

**DOI:** 10.1186/s12885-015-1006-6

**Published:** 2015-01-21

**Authors:** Philip Savage

**Affiliations:** BCCA Vancouver Island, 2410 Lee Avenue, Victoria, BC V8R 6V5 Canada

**Keywords:** Cancer, Chemotherapy, Apoptosis, Chemosensitivity, Meiosis, Gastrulation, VDJ, Hypermutation

## Abstract

**Background:**

A select number of relatively rare metastatic malignancies comprising trophoblast tumours, the rare childhood cancers, germ cells tumours, leukemias and lymphomas have been routinely curable with chemotherapy for more than 30 years. However for the more common metastatic malignancies chemotherapy treatment frequently brings clinical benefits but cure is not expected. Clinically this clear divide in outcome between the tumour types can appear at odds with the classical theories of chemotherapy sensitivity and resistance that include rates of proliferation, genetic development of drug resistance and drug efflux pumps. We have looked at the clinical characteristics of the chemotherapy curable malignancies to see if they have any common factors that could explain this extreme differential sensitivity to chemotherapy.

**Discussion:**

It has previously been noted how the onset of malignancy can leave malignant cells fixed with some key cellular functions remaining frozen at the point in development at which malignant transformation occurred. In the chemotherapy curable malignancies the onset of malignancy is in each case closely linked to one of the unique genetic events of; nuclear fusion for molar pregnancies, choriocarcinoma and placental site trophoblast tumours, gastrulation for the childhood cancers, meiosis for testicular cancer and ovarian germ cell tumours and VDJ rearrangement and somatic hypermutation for acute leukemia and lymphoma. These processes are all linked to natural periods of supra-physiological apoptotic potential and it appears that the malignant cells arising from them usually retain this heightened sensitivity to DNA damage. To investigate this hypothesis we have examined the natural history of the healthy cells during these processes and the chemotherapy sensitivity of malignancies arising before, during and after the events.

**Summary:**

To add to the debate on chemotherapy resistance and sensitivity, we would argue that malignancies can be functionally divided into 2 groups. Firstly those that arise in cells with naturally heightened apoptotic potential as a result of their proximity to the unique genetic events, where the malignancies are generally chemotherapy curable and then the more common malignancies that arise in cells of standard apoptotic potential that are not curable with classical cytotoxic drugs.

## Background

In the modern era of cancer therapies with designated targets and molecularly designed pathway inhibitors, the concept that crude DNA damaging cytotoxic chemotherapy agents could lead to successful treatment and the cure of some malignancies with minimal long term toxicity [1] would appear both old fashioned and unlikely. However the use of cytotoxic chemotherapy drugs to treat malignancies has been an integral part of cancer care since the 1950s [2] and in the treatment of a limited number of malignancies it has been spectacularly successful [3].

In the first 25 years of cytotoxic chemotherapy clinical drug development, there were dramatic advances in care that led to patients with a select number of relatively rare malignancies becoming routinely curable. By the end of the 1970s, the outlook for patients with gestational trophoblast tumours, testicular and ovarian germ cell tumours, acute leukaemia, Hodgkin’s lymphoma, high grade non-Hodgkin’s lymphoma and some of the childhood malignancies had been transformed with cure by then a realistic routine outcome [4].

With advances in drug delivery and supportive care, the majority of patients currently diagnosed with these rare malignancies can now expect curative treatment with the use of chemotherapy drugs that were all almost entirely developed before the 1980s. In contrast, despite the subsequent introduction of an additional 30 cytotoxic chemotherapy drugs and complex methods of delivery including high dose chemotherapy with stem cell rescue, the outlook for patients with the other more common types of metastatic cancers including breast, ovary, lung, prostate, colon, pancreas and melanoma remains one of disease control, improving life expectancy but without any significant chance of cure [4]. This divergent response to the same drugs used in the chemotherapy curable malignancies and those where the same cytotoxic chemotherapy drugs bring important benefits but not cure, remains one of the major challenges in clinical practice and cancer research [5-7].

It is apparent that the response to DNA damage from radiation therapy or chemotherapy can lead cells to proceed to either DNA repair or the induction of apoptosis [8]. The pathway taken in this divergent response is linked to the treatment dose and hence amount of DNA damage achieved [9] however it is clear that differing tumour types have dramatically differing thresholds for the effective induction of apoptosis as opposed to proceeding with DNA repair.

Over the past 30 years there has been with much research into this issue, looking at the postulated mechanisms of chemotherapy resistance and how to potentially overcome these barriers [10-12]. Historically the sensitivity and resistance of cancer cells to chemotherapy has been linked to the rate of cell division [13], the structure of the tumour cell DNA, the number of tumour cells and the subsequent risk of development of resistant clones [14,15], the presence of drug efflux pumps [16], reduction in DNA repair mechanisms [17] and for the curable cancers their relationship to primitive stem cells and a wide range of additional factors [12,18]. More recent data has raised issues regarding these concepts and the relationship between responses or resistance to chemotherapy and these previously established principles seems less secure [19-21]. From the clinical perspective these concepts of the determinants of chemotherapy curability can appear challenging, with the high levels of chemotherapy curability seen reliably in a selective number of tumours and the lack of a clear clinical relationship between the rate of growth of the chemotherapy curable tumours, which is frequently relatively slow and the often extreme bulk of the curable malignancies that appear to only rarely give rise to chemotherapy resistant clones.

In contrast, based on the key clinical characteristics of the chemotherapy curable malignancies, we have previously outlined a differing hypothesis behind the dramatically different outcomes to treatment between the rare chemotherapy curable malignancies and the other more common malignancies. We observe that each of the chemotherapy curable malignancies arises in a cell type that normally has a complex developmental path of differentiation into its mature progeny. During their development these cell types each undergo a specialised unique genetic process; nuclear fusion, gastrulation, meiosis, immunoglobulin/T cell receptor gene VDJ rearrangement, somatic hypermutation and class switching. These processes are each naturally intrinsically associated with heightened pro-apoptotic pathway activity [22,23]. The onset of these processes allows the normal healthy cell and its transformed malignant variant to have an exaggerated apoptotic response to both intrinsic DNA damage and also to chemotherapy induced therapeutic DNA damage. This response is both dramatically higher than that for normal cancer cells, but is also higher than that of the stem cells from which the malignant cell is derived.

It has previously been noted how the onset of malignancy during B lymphocyte development appears to leave the malignant cell ‘frozen’ at that developmental state [24,25]. Building on this hypothesis and the possibility that chemotherapy curable cancers are intrinsically different from normal tumours [26] this theory has been further developed for the wider group of chemotherapy curable malignancies. We have noted that the ‘frozen’ state also includes the up regulated pro-apoptotic processes and sensitivity that were naturally associated with the processes of DNA manipulation in action at the time of the development of the malignant phenotype [27].

In the clinical setting the large majority of malignancies arising at these key genetic points in these developing cells retain this dramatic sensitivity to DNA damage via the retention of their specialised pro-apoptotic pathways. This sensitivity can then be exploited with chemotherapy treatment despite the potentially long interval from the original malignant change through to diagnosis and treatment often many years later.

In this update we will review, from the clinical perspective with a focus on the phenomenon rather than the detailed mechanisms involved, the clinical and basic scientific data as to how this hypothesis may be applicable across the range of chemotherapy curable malignancies and consider how future therapeutic endeavours could exploit these processes.

## Discussion

### Review of metastatic malignancy chemotherapy curability

In 2015 it is perhaps challenging to understand quite the impact produced by the early success of cytotoxic chemotherapy treatment. The revolution in care that took place from 1955–1980 is perhaps the most dramatic that will be seen in cancer treatment development. In this period a number of malignancies that predominantly affected younger patients and were previously nearly uniformly fatal moved to becoming routinely curable, both in the major academic centres and also in routine clinical care [3,28].

In Table 1 the chemotherapy curable metastatic malignancies are listed along with representative treatment results and the chemotherapy drugs routinely used in current care. Of note, all but one of the drugs currently routinely used in the standard regimens employed in these chemotherapy curable malignancies were introduced to routine practice at least 30 years ago, with etoposide the last one added in 1982. These drugs were developed at a time when there was significantly less understanding about the metabolism, targets or pathway defects in cancer cells and the overall research budget only a fraction of the current one.

**Table 1:** **A summary of the unique genetic events associated with the chemotherapy curable malignancies**

Tumour type	Unique genetic event	Modern cure rate for metastatic disease	Standard drugs	Reference
Trophoblast tumours				
Post Mole GTT	Nuclear Fusion	100%	Methotrexate or Dactinomycin	[29]
Gestational choriocarcinoma	Nuclear Fusion	95%	Etoposide, Methotrexate Dactinomycin, Cyclophosphamide, Vincristine	[30]
PSTT	Nuclear Fusion	49%	Etoposide, Methotrexate Dactinomycin, Etoposide, Cisplatin	[31]
Germ cell tumours				
Testicular cancer	Meiosis	90%	Bleomycin, Etoposide, Cisplatin	[32]
Ovarian germ cell tumour	Meiosis	81%	Bleomycin, Etoposide, Cisplatin	[33]
Childhood malignancies				
Wilms	Gastrulation	80%	Dactinomycin, Vincristine, Doxorubicin	[34]
Neuroblastoma	Gastrulation	40–90%	Carboplatin, Etoposide, Cyclophosphamide, Doxorubicin	[35]
Ewings	Gastrulation	30%	Vincristine, Doxorubicin, Ifosfamide, Etoposide Busulphan Melphalan	[36]
Acute leukemia				
B ALL	VDJ rearrangement	Child 85%	Dexamethasone, Vincristine, Asparaginase Daunorubicin	[37,38]
		Adult 53%		
T ALL	VDJ rearrangement	Child 75%	Dexamethasone, Vincristine, Asparaginase Daunorubicin	[39]
AML	VDJ rearrangement	40%	Daunorubicin, Cytarabine	[40]
B Cell malignancies				
Hodgkin’s lymphoma	Somatic Hypermutation	80%	Doxorubicin, Bleomycin Vinblastine Dacarbazine	[41]
Diffuse large B cell lymphoma	Somatic Hypermutation	75% (GCB)	Cyclophosphamide, Doxorubicin, Vincristine, Prednisone, Rituximab	[42]
		40% (ABC)		
Burkitt’s lymphoma	Somatic Hypermutation	80%	Cyclophosphamide, Vincristine, Doxorubicin, Methotrexate, Ifosfamide	[43]
			Etoposide, Cytarabine	
T Cell malignancies				
ALK + ve anaplastic large cell lymphoma	V(D)J rearrangement	80%	Cyclophosphamide, Doxorubicin, Vincristine, Prednisone	[44]

There have been nearly 30 additional classical cytotoxic drugs licensed since the arrival of etoposide but none currently form part of the routine first line regimens for the chemotherapy curable malignancies [45]. Of the total of 78 new cancer treatment drugs of all therapeutic classes introduced during this time period, only one Rituximab, currently forms part of the routine treatment of a chemotherapy curable metastatic malignancy and then as an addition to therapy rather than a substitution of an earlier drug [42].

This data on the timeline of the key chemotherapy drugs used in the chemotherapy curable malignancies raises a key question that can be looked at in two differing ways; Why have there been no better drugs developed for treating these chemo-curable malignancies and extending curability to other malignancies developed since 1980, or alternatively what is different or special about these malignancies that made them so dramatically responsive to these early empirical chemotherapy drugs that the past 30 years of research and drug development has not been able to improve upon or extend to other tumor types?

Historical explanations for this extreme sensitivity to chemotherapy and the divergence in curability have been based on concepts including a higher growth rate for the curable cancers, the mutation of cells into drug resistant clones, the development of drug efflux pumps and the relationship of curable cancers to stem cells. However routine clinical observations indicate that the malignancies that are routinely chemotherapy curable occur in differing anatomical locations, present at differing ages and having differing clinical presentations and varying pace of growth. These can range from the very rapidly growing acute leukemias and gestational choriocarcinoma to the frequently slow growing, post molar pregnancy gestational trophoblast tumors, Hodgkin’s disease and testicular seminoma. Additionally the tumour types that are chemotherapy curable are more sensitive to chemotherapy drugs than their native stem cells and can in the case of B cell lymphoma have intermediary development stages and malignancies that are less sensitive to chemotherapy than in the further malignancies occurring later in B cell development.

These clinical observations indicate that the malignancies that are chemotherapy curable each arise in cells that are associated with physiologically unique genetic events and that the highly up regulated pro-apoptotic processes that naturally accompany these events are maintained in the associated malignancies.

The unique genetic processes occurring in the chemotherapy curable malignancies are nuclear fusion for molar pregnancies, gestational choriocarcinoma and placental site tumors, gastrulation for the rare childhood malignancies, meiosis for the testicular and ovarian germ cell tumours, VDJ rearrangements for B cell and T cell ALL, acute myeloid leukemia and anaplastic large cell lymphoma and immunoglobulin gene somatic hypermutation for Hodgkin’s lymphoma and diffuse large B cell lymphoma (DLBCL).

Our hypothesis is that these chemo-curable malignancies are a unique group of malignancies that whilst arising at different time points and from differing cells lines, all share the presence of hugely up regulated pro-apoptotic pressures that were present in these cells as part of their natural activity during a normal but unique genetic activity occurring in these cells at the time of the development of the malignant phenotype. As a result of the malignant change the cells appear to be held developmentally frozen and poised to potentially naturally undergo apoptosis in response to either natural or therapeutic DNA damage. Within this scenario the chemotherapy drugs, particularly when used in combination exploit this situation, producing massive levels of apoptosis that the newer drugs are unable to improve significantly on. The links between these genetic events, the clinical presentations and treatment outcomes for the differing groups of malignancies are discussed below.

### Gestational Trophoblast Tumours [GTT]

#### Post molar pregnancy-GTT

The process of nuclear fusion, the joining together of the nucleus of the oocyte and that of the sperm, is complex and frequently ends in failure rather than the successful creation of a viable embryo. The complex process also includes the second meiotic division of the oocyte, a process that happens a number of decades after the first meiotic division which occurred in utero. Oocytes that are not fertilised appear to die via apoptosis [46] with the progression to this natural process being inhibited by successful fertilisation [47]. Similarly in spermatogenesis, apoptosis is a routine part of the process with estimates of losses of 70% reported [47]. More recent data has indicated that normal healthy sperm have active apoptotic characteristics and are destined to naturally die an apoptotic death, and hence avoid a potential inflammatory and immunogenic death, unless rescued by fertilising the egg [48].

In the successfully fertilised eggs, appreciable levels of apoptosis are still exhibited and many zygotes, predominately those with genetic abnormalities, die an apoptotic death shortly after fertilisation [49,50]. Additionally apoptosis is actively involved in balancing the numbers of cell within the blastocyst and inner cell mass [51] and the second polar body, the product of the second meiotic division of the oocyte that is produced at the time of fertilisation, is also lost by undergoing apoptosis [52,53].

The pro-apoptotic pressures appear to remain active initially after nuclear fusion and the newly fertilised human egg remains extremely sensitive to DNA damaging agents. In response to very low doses of chemotherapy the cells of the new fertilised egg and developing embryo readily undergo apoptosis. In ectopic pregnancies a single 50–100 mg dose of methotrexate is usually adequate to kill the cells [54]. Similarly when used to induce a medical termination, similar low doses of methotrexate are also able to terminate pregnancies through to 8 weeks gestation with near 90% effectiveness [55]. In contrast by the end of the first trimester the cells are generally resistant to chemotherapy and maternal malignancies can be treated with full dose combination chemotherapy without any detectable detriment to the foetus or placenta [56]. In post molar pregnancy gestational trophoblast tumours, it appears that this extraordinary sensitivity to DNA damage that is associated with nuclear fusion, can remain in place virtually indefinitely and so gives this diagnosis its great sensitivity to chemotherapy [29].

Molar pregnancies have characteristic abnormal genetic structures with partial molar pregnancies having two sets of paternal chromosomes and one set of maternal chromosome [57] and complete molar pregnancies having two sets of paternal but no maternal chromosomes [58]. As a result, the cells of the molar pregnancy are genetically and structurally abnormal from the point of conception and we would hypothesise that in molar pregnancies the natural apoptotic processes existing at the time of nuclear fusion do not become down regulated, as they do in a normal pregnancy, despite the multiple division of the cells and growth of the molar tissue.

**Figure 1:**
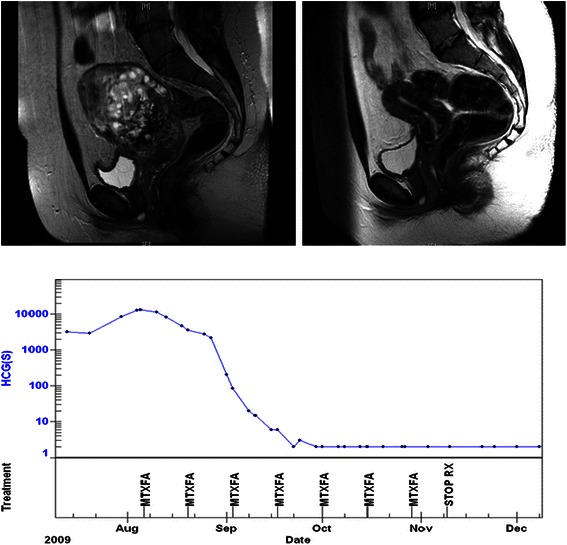
**Post molar pregnancy gestational trophoblast tumour.** Pre and post treatment MRI scans of the pelvis showing a large uterine mass prior to treatment and a normal scan at completion. The treatment graph shows the fall and normalisation of the hCG level with low dose methotrexate chemotherapy treatment. The patient has completed treatment, gone on to have a healthy baby and has been cured of this malignancy.

Low dose methotrexate was first successfully used in the treatment of post molar pregnancy gestational trophoblast tumours in 1956 [2] and protocols based on low dose methotrexate remain the standard of care at most treatment centres [29,59]. A routine example of the rapid response treatment and curative outcome for a patient with post molar pregnancy gestational trophoblast tumours is shown in Figure 1. Overall 100% of patients with post molar pregnancy gestational trophoblast tumours can be cured with chemotherapy, with the majority only requiring therapy with doses of methotrexate that would be sub-therapeutic and have little activity in other malignancies [29].

#### Gestational Choriocarcinoma and Placental Site Trophoblast Tumour [PSTT]

Gestational choriocarcinoma is a rare malignancy and has an incidence in the range of approximately 10–20 cases per million conceptions [60]. The typical presentation of gestational choriocarcinoma is of a rapidly growing, hCG producing malignancy occurring in women after a normal pregnancy with frequently widespread metastatic spread with the CNS, lung and liver common disease sites. Despite the disseminated pattern of metastatic spread and the frequent rapid growth of the tumour cells the large majority of cases of choriocarcinoma are routinely curable with combination chemotherapy [30].

**Figure 2:**
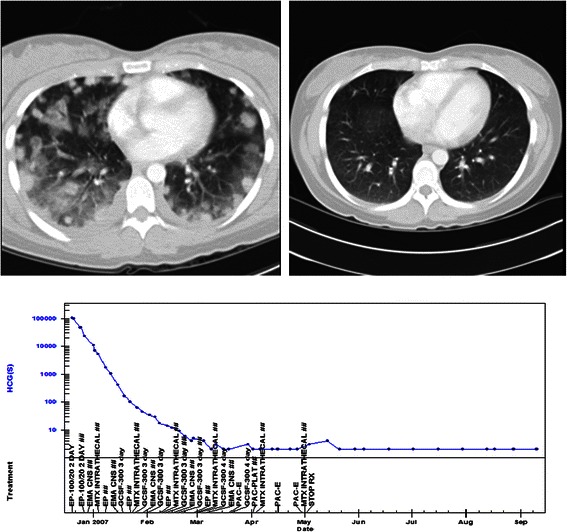
**Gestational choriocarcinoma.** Pre and post treatment CT scans of the chest showing multiple lung metastases and pulmonary haemorrhage at diagnosis, 12 weeks post partum and a normal scan at completion of chemotherapy. The treatment graph shows the fall and normalisation of the hCG level with combination chemotherapy treatment. The baby was unaffected by the illness, the patient has completed treatment and gone on to have another healthy baby and has been cured of this rare malignancy.

Clinical data from the care of a patient with gestational choriocarcinoma are demonstrated in Figure 2, showing the prompt hCG tumour marker response and complete radiological response of the multiple lung metastases to chemotherapy treatment. The patient remains well nearly 10 years later, has had a further healthy child and has a negligible risk of relapse.

In gestational choriocarcinoma the malignant cells arise from previously genetically normal cells of a normal conception that have successfully moved through fertilisation and nuclear fusion. In embryogenesis the differentiated trophoblast cells start to develop at approximately 3 days post fertilisation, as the cells of the morula differentiate into the outer trophoblast cells and the inner cell mass. Whilst the cells of the inner cell mass go onto form the embryo, it is the cytotrophoblastic cells in the outer component that can become transformed and appear to be the cell of origin of gestational choriocarcinoma [61].

Placental site trophoblast tumour [PSTT] is a rarer diagnosis with an incidence of approximately 2–5 cases per million pregnancies. In contrast to choriocarcinoma, PSTT develops from a transformed cell that arises later in the development of the placenta. The cells of origin of PSTT are the implantation site intermediate trophoblast cells [62,63] which develop later in the pregnancy becoming apparent at approximately 13–16 days post conception [R Kurman pers comm].

Compared to post molar pregnancy GTT, the cellular points of origin of both gestational choriocarcinoma and PSTT are significantly different occurring from a normally fertilised egg and from further along the development route after the initial fertilisation and nuclear fusion. This increased temporal distance from the unique genetic event of nuclear fusion parallels the clinical observation of the rising intensity of chemotherapy needed to cure these tumours. Overall gestational choriocarcinoma is usually curable with chemotherapy but single agent low dose methotrexate is rarely effective [64] and more intensive combination regimens are needed to give curative treatment for most patients [30]. In PSTT the malignant cells arise from intermediate trophoblast, a cell type that is further removed from the nuclear fusion event. In this diagnosis, combination chemotherapy can be curative in many patients with metastatic disease but the overall cure rates of 49% are significantly lower than in post molar pregnancy gestational trophoblast tumours or gestational choriocarcinoma [31].

In choriocarcinoma and PSTT, in addition to the importance of the genetic distance the tumour arises from the nuclear fusion event, the interval from the antecedent pregnancy to the time of diagnosis and treatment appears to have a significant impact on chemotherapy curability. In choriocarcinoma, patients with a longer interval from the antecedent pregnancy to presentation have an enhanced risk of refractory disease and treatment failure [65] and whilst cases of PSTT presenting with an interval of less than 48 months have a good prognosis those with a longer interval are rarely cured [31]. It is unclear if these changes in curability mark the loss of the pro-apoptotic pathways occurring with the increasing interval from the antecedent pregnancy or if the cases of choriocarcinoma or PSTT arising later in the development of the trophoblast and hence more distant from nuclear fusion have both a more slow growing natural history, later presentation and also a poorer response to chemotherapy.

### Childhood malignancies

The successful treatment of the rare childhood malignancies including Ewing’s sarcoma, Wilms tumours, osteosarcoma and neuroblastoma has been one of the great successes of paediatric oncology. Chemotherapy has been effectively used in these diagnoses since the 1950s and now overall the majority of children with these rare malignancies can be cured [66,67].

The incidence of Ewing’s sarcoma is approximately 10 cases per million for those aged 10–19 [68] and for patients with localised disease the cure rate with combined modality treatment is 71–94% [69] For patients with metastatic disease at presentation approximately 30% will be cured using treatment based on the classical cytotoxic chemotherapy drugs vincristine, doxorubicin, ifosfamide, etoposide and busulphan and melphalan [36]. Similarly Wilms tumour is rare with an incidence of 1 in 10,000 children and characteristically presents with a renal mass [70]. The use of combined modality treatment including adjuvant chemotherapy has transformed the prognosis of this rare condition, with patients with low stage disease having cure rates in excess of 90% [71], whilst for the patients presenting with metastatic disease chemotherapy treatment predominantly using vincristine, dactinomycin, and doxorubicin can result in cure rates of approaching 75% [34,72].

Osteosarcoma is a rare malignancy of childhood with an estimated incidence of 4 cases per million between the age of 0–24 [73]. The impact on chemotherapy in this rare malignancy has been most marked in the success of adjuvant therapy after surgery, however for young patients with metastatic disease at presentation chemotherapy treatment can be curative. A recent SEER overview indicates for young patients likely cure rates of 80% for those presenting with localised disease, 65% for regional disease and 25% for patients with metastases [73].

A further childhood cancer, neuroblastoma occurs with an incidence of 10 cases per million children [74]. Chemotherapy treatment using classical cytotoxic drugs including cyclophosphamide, doxorubicin, carboplatin, etoposide produces a cure rate of 95% for patients with good prognosis metastatic disease whilst patients with localised disease have cure rates approaching 100% [35].

Historically, there has been debate regarding the cell of origin of these malignancies, now there is an increasing body of evidence that Ewing’s sarcoma, Wilms tumours and osteosarcoma arise from mesenchymal stem cells [75-78], whilst the likely cell of origin of neuroblastoma are the fetal neuroblast cells from the neural crest [79,80]. These two cell types share significant characteristics and it has been hypothesized that the two types are actually two phenotypes of a common cell of origin [81].

Whilst the exact point of origin of the malignancies remains uncertain and there is relatively little information on the molecular pathways involved on both oncogenesis and chemosensitivity, these tumors all appear to arise from very primitive embryonal cells rather than more differentiated mature cells.

In Ewings tumours there is the characteristic translocation EWS/ETS transformation which leads to the transformation and the malignant phenotype [75]. More recent publications have indicated that some of the key molecular components in Ewings including EZH2 are already present at gastrulation and can prevent cellular differentiation [82]. Other recent work has suggested that Ewings cells are derived from primordial streak early mesoblasts [83] or neural crest derived stem cells [84] which is in keeping with the original observations from Ewing in 1921 [85].

In line with the theme that the other chemotherapy curable malignancies have a close relationship with unique genetic events, we can hypothesis that the chemotherapy curability of the childhood malignancies is linked to the close relationship of these cells with the process of gastrulation. This process occurs in human embryos at approximately day 16 when the single layer of the blastocyst segregates into the three new germ layer components of the ectoderm, mesoderm, and endoderm. The ectoderm goes on to give rise to the skin, nerves and neural crest cells, the mesoderm the bones, connective tissues and urogenital system and the endoderm the gut and associated organs. During the process of gastrulation there is cellular differentiation and a dramatic increase in cell proliferation and growth. During this period there is a shortened cell cycle, which may preclude cell cycle arrest and full DNA repair in response to DNA damage. The process of gastrulation is accompanied by an increased sensitivity to DNA damage, with animal data indicating that the apoptotic response to DNA damage can be up to 15 fold higher during gastrulation than before or after the process [86].

Whilst there is little direct data to examine, we can hypothesise that these cancers develop a malignant phenotype during or soon after gastrulation and subsequently become frozen at their development point in a similar fashion to the B cell malignancies and so still retain some of the heightened apoptotic potential associated with the gastrulation process. As a result in a similar situation to the patients with the other chemotherapy curable malignancies these pro-apoptotic pressures are then exploited therapeutically with the chemotherapy induced DNA damage resulting in highly effective apoptosis and cell kill and the high cure rates observed.

### Germ cell malignancies

**Figure 3:**
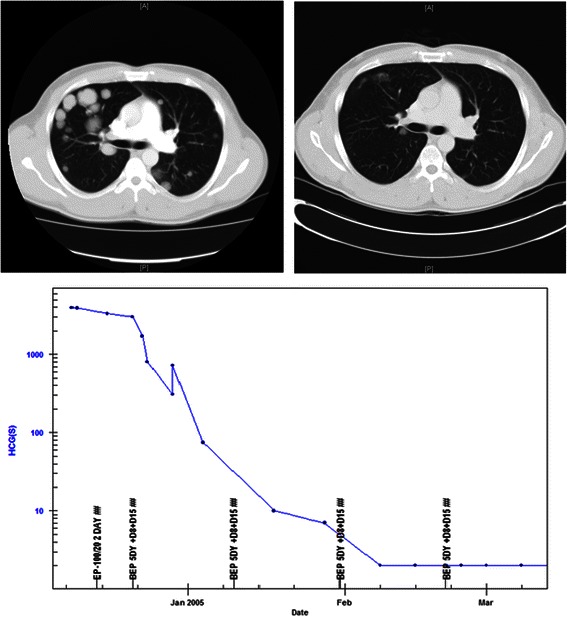
**Advanced testicular cancer.** Pre and post treatment CT scans of the chest showing multiple pulmonary lesions at diagnosis and virtual resolution of the lesions by the time of completion of chemotherapy. The treatment graph shows the decline and normalisation of hCG levels with BEP chemotherapy treatment. Nine years on the patient is in a complete remission, and is almost certainly cured. The lung lesions resolved completely with longer follow up.

Metastatic testicular cancer has been potentially curable with chemotherapy since the 1960s [87] and modern treatment now brings overall cure rates exceeding 80% [88,89]. A routine example case of a patient cured of intermediate risk testicular cancer is shown in Figure 3. Similarly in women, advanced ovarian germ cell tumours, whilst much rarer, are also effectively treated with combination chemotherapy with overall cure rates of approximately 80% [33]. In both diagnoses the most widely used regimen of BEP comprises bleomycin, etoposide, and cisplatin, which are three conventional cytotoxic drugs all introduced into routine care prior to the mid-1980s [32,33].

The majority of testicular germ cell tumours arise from areas of the pre-malignant precursor CIS [carcinoma in situ, also referred to as IGCNU or gonocytoma in situ] [90,91]. This premalignant condition is believed to arise from fetal gonocytes, with arrested development, that persist inappropriately in the post natal testis rather than making the standard progression to become quiescent pre-pubertal pre-spermatogonia [92]. The pathway to the onset of malignancy and development of testicular cancer is an area of relative scientific and biological complexity [93]. The overall process of meiosis includes a number of additional key complex genetic events; including the repair of double stand DNA damage [94] and the erasure of imprinting via genome wide demeythylation [95], a situation that persist in CIS cells [96].

**Figure 4:**
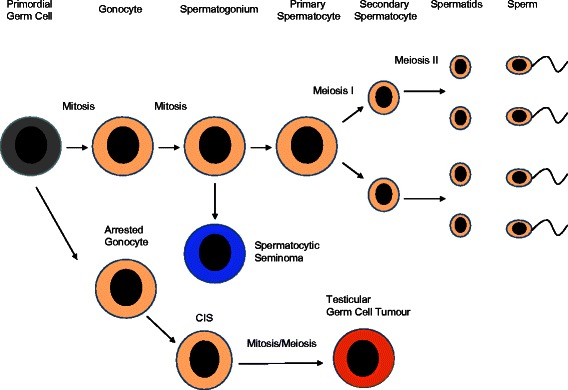
**Spermatogenesis and cancer development.** The normal and malignant development of testicular germ cells. The normal progression from the primordial germ cell to the production of sperm is shown in the upper pathway. Testicular germ cell tumours develop via CIS from arrested gonocytes that have an impaired mitosis/meiosis switch. Spermatocytic seminoma the malignancy arising from normal pre-meiotic spermatogonia is characteristically chemotherapy resistant.

A simplified overview of the development of germ cells and spermatogenesis can be summarised in the following steps as shown in Figure 4. In the embryo at approximately at 21–28 days gestation the primordial germ cells arise from the proximal epiblast adjacent to the extra-embryonic ectoderm [97]. During weeks 4–8, the primordial germ cells migrate along the autonomic nerve fibres to the genital ridge [98]. Up to this point, the development processes are shared in both male and female embryos, however in female embryos, the effect of retinoic acid from the mesonephric duct at week 11, induces the commencement of meiosis [99]. At this point meiosis proceeds only through to the meiotic prophase I with the cells becoming oocytes enclosed in primordial follicles towards the end of the first trimester [100]. In contrast in males at the start of gonadal development at approximately 6 to 7 weeks gestation, the action of sex-determining region Y (SRY) leads to Sertoli cell differentiation and production of cytochrome p450 26B1 which degrades retinoic acid [101] which inhibits the progression to meiosis. Additional newer data also suggests that other parallel pathways including the expression of DMRT1 and STRA8 by the Sertoli cells may also play a key role in this inhibition [102].

As a result of these processes in pre-pubertal males the healthy gonocytes (pro-spermatogonia) are prevented from entering meiosis and continue to divide mitotically and then normally mature into pre-spermatogonia [103]. In contrast in cases of CIS the gonocytes are held in development as embryonic germ cells in the pre-pubertal and adult testis and do not follow the normal progression to pre-spermatogonia [104].

In health the rise in testosterone levels at the onset of puberty alters the balance between the inhibition and promotion of meiosis and spermatogonia A start the processes to produce sperm, dividing and giving rise to the spermatogonia B and these then form the spermatocytes that produce the sperm.

In the developmentally blocked CIS cells the onset of puberty also leads to enhanced activity and the rising risk of developing a malignant phenotype [105]. Whilst historical scientific data suggested that CIS cells did not arise from cells associated with meiosis [92] more recent data indicates that the balance of the mitosis-meiosis switch within the CIS cells is significantly altered at puberty with pressure for both processes to occur. Recent publications indicate that the meiosis inducing proteins STRA8, SCP3 and DMC1 are present in CIS cells whilst at the same time the meiosis inhibiting NANOS2 and CYP26B1 regulators are also expressed [102]. As a result there are concurrent pressures in the cells for both mitosis and meiosis to be induced, a process which helps the malignant phenotype develop [106] but also fortunately leads to the developing malignant cells having their great sensitivity to chemotherapy.

The induction of apoptosis is a routine event in healthy spermatogenesis and it is estimated that 75% of sperm production undergoes apoptosis [47]. A number of clinical observations lend support to the hypothesis that it is the onset of meiosis in the CIS/tumour cells which is linked to the development of the extreme sensitivity to chemotherapy via DNA damage and the induction of apoptosis, rather than their proximity to the spermatogonial stem cells that drive the system.

Data from studies in which the testis has been exposed to low doses of radiation demonstrate that the radiation sensitivity of the differing stages of cells involved with spermatogenesis varies significantly along the developmental pathway. The cells that are most sensitive to DNA damaging agents are the type B spermatogonia that are undergoing going mitosis in preparation for meiosis and the meiotic primary spermatocytes, whilst the post meiosis spermatids and the spermatogonial stem cells are much more resistant [107-109]. This differential toxicity is supported from data from cancer patients treated with chemotherapy or radiotherapy that indicates that sperm counts fall significantly by 10 weeks but do not reach azoospermia until 12 weeks post exposure, a timescale in keeping with a production block at the level of the type B spermatogonia [110-112].

In oncology patients treated with chemotherapy the recovery of sperm production generally occurs as the stem cell spermatogonia recover and restart the process of spermatogenesis and repopulate the intermediate cells stages [109]. This process can take between 12 weeks and 2 years dependent on the chemotherapy drugs administered.

From this animal and clinical data it is apparent that whilst the cells involved in preparing for and undergoing meiosis are rapidly killed by DNA damaging agents, their predecessors the spermatogonial stem cells are not killed and these stem cells are with time able to repopulate the whole production system. This finding supports the hypothesis that the sensitivity to chemotherapy is not an intrinsic characteristic of the stem cells themselves, but a characteristic acquired by the cells whilst preparing for and undergoing meiosis. Additional support to the hypothesis that the pre-meiotic spermatogonial stem cells are resistant to chemotherapy comes from recent data looking at the cell of origin of the chemotherapy resistant malignancy spermatocytic seminoma. This rare malignancy is characterised by a high degree of resistance to chemotherapy [113] and recent work has confirmed that the cell of origin of this malignancy is the spermatogonial stem cell [114]. The spermatogonial stem cells have not made the progression to meiosis and the malignant cells arising from them show much greater resistance to chemotherapy compared to either healthy spermatogonia cells undergoing meiosis and or to the CIS derived testicular germ cell tumours.

#### Ovarian germ cell tumors

Malignant ovarian germ cell tumors (mOGCT) are rare, occurring at less than 10% the rate of testicular cancer with a peak incidence of approximately 1 case per 100,000 women aged 15–19 [115]. The most frequent pathological type is dysgerminoma, which is similar to seminoma in men whilst non-dysgerminomas have a similar range of pathologies as non-seminomatous germ cell tumours have in men [116].

**Figure 5:**
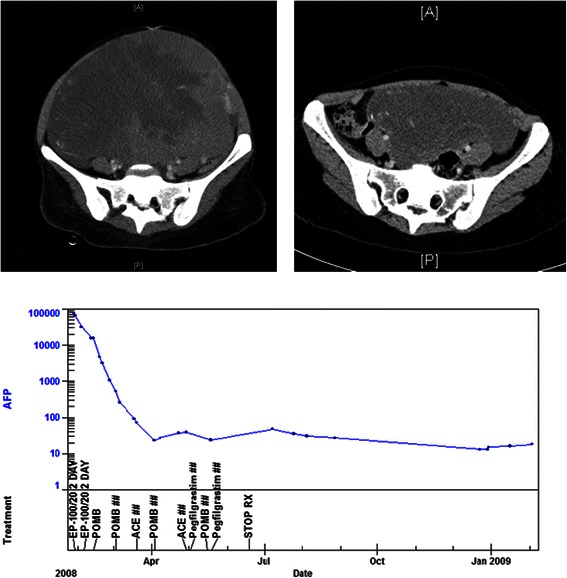
**Ovarian germ cell tumour.** Pre and post treatment CT scans of the pelvis showing an enormous ovarian germ cell tumour filling the abdomen and pelvis prior to chemotherapy treatment. The residual mass in the post treatment scan was resected and was composed entirely of necrotic material. The treatment graph shows the tumour produced hCG level falling to normal within the first month of treatment. The low level of pituitary hCG production secondary to amenorrhea resolved with the restarting of menstruation 12 months after completion of treatment.

Whilst early stage ovarian germ cell tumours can be managed by surgery and surveillance based on tumour markers and imaging [117], stage II and more advanced tumors are treated with combination chemotherapy treatment [118]. Similarly to testicular cancer, chemotherapy with cisplatin based regimens such as BEP and POMB-ACE can bring dramatic responses to treatment and cure is the most likely outcome even for patients with widespread metastatic disease [33]. As an example of the high degree of chemosensitivity in this diagnosis the imaging of a patient presenting with very large mOGCT and cured with chemotherapy is demonstrated in Figure 5.

In women the development pathways of oocytes and the risks and mechanisms for malignant change have many similarities to testicular germ cell tumours in men. In the fetal ovary the germ cells under the influence of retinoic acid commence meiosis and become fixed in meiotic prophase 1 with the oocytes going on to form the primordial follicles [119]. In health the oocytes are held at the stage of the first meiotic division, which took place in the developing fetus, until ovulation a number of decades later, when they complete the first meiotic division and then again stop in metaphase in the second meiotic division at the time of the release of the egg from the ovary. If the egg is fertilised there is then completion of the second meiotic division and the release of the polar body after fertilisation [101].

Whilst in testicular germ cell tumours, the cell of origin of the malignancy is CIS, in mOGCT the likely cell of origin are gonadoblastoma cells, that are characterised by containing fetal germ cells mixed with immature Sertoli and granulosa cells and have many similarities to testicular CIS cells [120]. Gonadoblastomas are only found exceptionally rarely in healthy ovaries with their development linked to Sertoli cell underdevelopment and an environmental imbalance in the development of the maturing ovary but are more common in women with disorders of sex development [121]. It is postulated that some of the germ cells within the gonadoblastoma, may have postponement or failure of the onset of meiosis and these cells could continue to divide mitotically [122].

The exact pathways for the malignant transformation in gonadoblastoma are complex but it is apparent that these cells retain their fetal phenotype and are susceptible to the development of malignancy [123]. In disorders of sex development patients with dysgenetic gonads and Y chromosomes, the activity of the TSPY gene [testis specific protein Y] in the gonadoblastoma locus appears to act as an oncogene [122]. Of interest in the healthy testis this protein is expressed in meiotic prophase I and plays a role in chromatin organisation and the progression of the prophase I and meiotic divisions [124,125].

In women with an XX genotype, ovarian germ cell tumors are rare and the exact process of oncogenesis in the absence of Y chromosome proteins is less clear. However in that gonadoblastoma cells are immature germ cells with a fetal phenotype in an adult ovary it is possible that the route to both malignant transformation and the extreme chemotherapy sensitivity seen in mOGCT could have similarities to the disruption of the meiotic mitotic switch seen as in CIS and testicular germ cell tumours [123].

The outlook for women treated for mOGCT is positive with cure rates in excess of 80%. Whilst the chemotherapy treatment is usually able to kill all of the tumour cells, however the healthy oocytes are mainly undamaged and the large majority of women usually recover their menstrual cycle after chemotherapy [126,127].

Overall in mOGCT it appears that malignant cells that are naturally highly sensitive to DNA damage arise from the gonadoblastoma, whilst the cells in the ovarian follicles that are in a halted meiosis (for decades) are relatively resistant. This finding again lends support to the hypothesis that it is the onset of active meiosis, rather than the relationship with the stem cell that is linked to the chemotherapy sensitivity and curability.

### B cell malignancies

**Figure 6:**
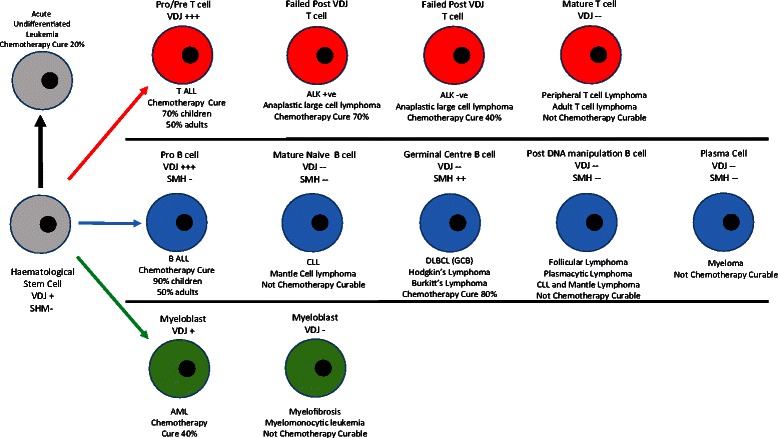
**Genetic events and chemotherapy sensitivity in malignancies derived from the haematological stem cells.** The curability with chemotherapy is shown to coincide with either VDJ gene rearrangement or somatic hypermutation. Malignancies occurring in cells not undergoing either of these process, are characteristically non chemotherapy curable.

The complex natural history of B cell development involves progression from the haematopoietic stem cell (HSC) through to final maturity via a number of key stages including pre B cell, marginal zone, germinal centre cell, mature B cell and plasma cell as shown in Figure 6. The malignancies that arise at differing points along this development have significantly different levels of response and curability with chemotherapy treatment. Acute lymphoblastic leukemia (ALL), diffuse large B cell lymphoma, Hodgkin’s lymphoma and Burkitt’s lymphoma are all routinely curable, whilst acute undifferentiated leukemia, chronic lymphocytic leukemia (CLL), mantle cell lymphoma, follicular lymphoma and myeloma are generally incurable with chemotherapy.

The variation in chemotherapy curability across the malignancies arising during B cell development shows a relatively low cure rate in the malignancy arising from the stem cells, but significant rises with some of the later cell types. This suggests that the overwhelming sensitivity to chemotherapy is a characteristic that is not present in the native haematopoietic stem cells, but is acquired for ALL, lost for marginal zone and mantle lymphomas, reacquired for diffuse large B cell lymphoma and Hodgkin’s lymphoma and then finally lost again later in B cell development. This pattern suggests that processes aside from the proximity to the stem cell, or the rate of cell growth, are responsible for the chemotherapy curability rather than the chemotherapy curability being simply as a result of developmental proximity of these malignancies to the stem cells themselves or the rate of growth.

It has previously been described how the onset of the malignant phenotype can freeze B cells at a point in their development process leading to the wide range of B cell malignancies from acute undifferentiated leukemia to myeloma [24,25]. From a clinical perspective it would also appear that the varying B cell malignancies of acute undifferentiated leukemia all the way through to myeloma also retain their then current natural level of apoptotic potential associated with the unique genetic processes of; VDJ rearrangement, somatic hypermutation and class switching that were occurring at the time of malignant transformation [27]. The degree of apoptotic sensitivity that is associated with these processes appears to remain in place in these malignancies, despite the numerous cell divisions and passage of potentially years from the onset of malignancy in a single cell through to the eventual time of diagnosis and treatment.

#### A brief overview of the natural history, development and genetic events in B cell development

B cell development commences with the hematopoietic stem cells that develop into pro B cells in the bone marrow. In the pro B cell the processes of VDJ rearrangement of the immunoglobulin heavy chain gene and VJ rearrangement of the light chain genes take place. These processes involves double strand DNA breaks in the immunoglobulin genes by the RAG proteins, at areas linked to the recombination signal sequences [128,129], which are then repaired by DNA repair mechanisms including non-homologous end joining (NHEJ) [130,131]. However the process recombines DNA randomly and only approximately one third of recombinations are successful and cells that fail to recombine and make a functional B cell receptor are lost via apoptosis [132,133].

The process of VDJ rearrangement activity in B cells commences at low levels in HSC and progenitor B cells and then has a peak of activity in pro B cells, declines in pre B cells and is absent in mature B cells [134]. Of interest the activity of the RAG systems that produce VDJ rearrangement starts activity significantly prior to the VDJ rearrangement events [135] and is not limited just to the haematopoetic stem cells destined to become B and T cells. There is also significantly VDJ activity occurring early in the development of hematopoietic stem cell that go on to the pathway of myeloid differentiation with VDJ rearrangement present in many cases of AML [136] and also occurring later in development prior to acute lymphoid blast crisis in CML [137].

B cells that have successfully recombined their immunoglobulin genes and have a functional B cell receptor turn off the VDJ system and leave the bone marrow as mature naïve B cells [138,139], whilst those that have an unsuccessful rearrangement perish by neglect and undergo apoptosis [140]. The next stage in B cell development is antigenic contact that triggers the germinal centre reaction that includes the two additional DNA modification steps of somatic hypermutation, followed by immunoglobulin class switching.

These processes are mediated by activation induced cytidine deaminase (AID) that produces mutations, insertions and deletions within the immunoglobulin hypervariable regions during somatic hypermutation [141]. Following somatic hypermutation the germinal B cell then undergoes class switching again using AID to cut the DNA and the NHEJ enzyme complex to repair the breaks [142,143].

These complexes processes of immunoglobulin gene VDJ rearrangement and somatic hypermutation are recognised to be associated with the induction of lymphomagenesis, with VDJ rearrangements particularly linked to malignancy associated translocations including t [8,14], t[14:18] and BCL2 [144-147], whilst somatic hypermutation is linked to the myc translocations seen in Burkitt’s lymphoma [148]. Despite this significant risk of oncogenesis occurring from immunoglobulin gene genetic rearrangement mechanisms, it appears from an evolutionary viewpoint, that the benefits of an adaptive immune system appear to outweigh the risks of malignancy associated with the process [149].

Whilst the exact timeline and cell of origin of some types of B cell malignancy are yet to be fully confirmed, there is a general consensus on most of the key types. The relationship between the cell of origin, the unique genetic processes and chemotherapy curability is demonstrated in Table 1 and discussed below.

#### Pre VDJ rearrangement, pre somatic hypermutation malignancies

During cytotoxic chemotherapy treatment the early progenitor stage haemopoietic stem cells are significantly affected by treatment but with standard dose chemotherapy these cells are not killed off and after an interval, stem cell function is fully recovered.

Malignancies arising from the primitive haemopoietic stem cells are very rare and relatively poorly characterised. Acute undifferentiated leukemia is believed to arise from the undifferentiated haematopoietic stem cells but a number of studies which have examined the status of the immunoglobulin and T cell receptor genes in this malignancy, indicate that a significant number of these cases have already commenced VDJ rearrangement [150]. However, overall less than 50% of cases have started VDJ rearrangement and a smaller proportion T cell receptor V[D]J rearrangement and the majority of cases of acute undifferentiated leukemia still have germ line unrecombined VDJ genes [150].

Acute undifferentiated leukemia arising from the haemopoietic stem cells has a poor prognosis despite modern treatment with intensive chemotherapy. Overall long term survival rates of approximately 20% have been reported [150] and the recent US SEER data indicates a 5 year survival rates of 65% for children, 35% for adults aged 20–39 but only 3% for those aged 60+ [151]. Taken together the cure rates from the malignancies that actually arise from the haemopoietic stem cells, are significantly lower than for the B and T cell acute lymphoblastic leukemias that arise at the next development stage, where the process of VDJ rearrangement is more fully active.

#### VDJ rearrangement associated malignancies

In contrast to acute undifferentiated leukemia, B cell ALL arises from pro B cells that are undergoing VDJ rearrangement in the bone marrow but are yet to proceed on to somatic hypermutation [133,152-154]. The treatment for B ALL patients centres on the use of classical cytotoxic chemotherapy drugs used with a high degree of dose intensity. The most frequently used regimens include dexamethasone, vincristine, asparaginase and daunorubicin, and the successful treatment of childhood B ALL has been one of the great success stories of malignant haematology [155]. Recent data from the US SEER series indicates that the cure rates range from >90% in children and teenagers to approximately 50% for those occurring in adults but falling to only 10% in the elderly [151].

The prognosis of B ALL diagnosis is significantly better than for those with more primitive diagnosis of acute undifferentiated leukemia that arises from the haemopoietic stem cells, the cell type preceding it in B cell development. This finding showing an increase in cure rates with differentiation is in keeping with the impact of the onset of VDJ rearrangement and contrary to that expected if proximity to stem cell function is the key contributor to chemotherapy curability.

Of interest the cure rates of B ALL vary distinctly with the age of the patient, ranging from near uniform cure in children down to only 10% in elderly patients [151]. Traditionally this decline with age has been ascribed to differing intensity of chemotherapy treatments, unfavourable mutations and worse prognosis presentation [156,157]. However, in line with the hypothesis that the overall sensitivity to chemotherapy in B ALL is a product of the mechanisms and associated apoptotic potential of VDJ rearrangement, it is also possible that the reduction in chemotherapy sensitivity and hence cure rates is related to the major decline in VDJ recombinase activity and potentially related pro-apoptotic potential seen in early B cells with increasing patient age. Recent data has demonstrated that the activity of the B cell VDJ system changes significantly with the age of the patients, with the overall activity levels of RAG1 and other key components falling dramatically from toddlers to middle aged adults giving a similar pattern to the fall in B ALL cure rates with increasing age [158]. Alongside this an number of key B cell signalling and B cell receptor proteins are expressed at only around 20% of the childhood level in adults [159].

#### Post VDJ rearrangement, pre somatic hypermutation associated malignancies

The malignancies that arise from B cells that have completed VDJ rearrangement but not undergone somatic hypermutation include mantle cell lymphoma and chronic lymphocytic leukemia [CLL]. In these diagnoses the majority of cases contain immunoglobulin genes that have successfully undergone VDJ rearrangement but not commenced somatic hypermutation [160,161]. In both of these diagnoses chemotherapy treatment brings major responses and remissions but they are not currently regarded as being curable.

Of interest approximately 20% of cases of mantle zone lymphoma and 50% of CLL have a degree of somatic hypermutation of their immunoglobulin genes and these cases are linked to a significant improvement in response to but not cure with chemotherapy treatment [162,163]. In mantle cell lymphoma the impact on the hyper-mutated status of the variable domain is significant as those with mutated genes have a 5 year event free survival of 75% vs only 23% for those with classical unmutated Mantle cell lymphoma [164]. In CLL an early study demonstrated dramatically divergent median patient survival dependent on the IGH mutation status of 95 months with unmutated IGH genes and 293 months for those with mutated genes. [162]. More recent studies using ZAP70 expression as a surrogate for IGH mutational status indicate similar positive results with patients with mutated IGH receiving more modern therapies [165]. At the molecular level it is apparent that in the in the cases of CLL that have evidence of SHM activity and mutated variable regions, serial analysis from CLL patients suggests that the SHM process is no longer on going during the clinical stages of the diagnosis [166].

#### Somatic hypermutation associated malignancies

The processes of somatic hypermutation takes place predominantly within the lymph node germinal centre and involves the action of activation induced cytidine deaminase [AID] on cells that have a successfully recombined IGH gene [167]. The action of AID is focused predominantly on the variable regions of the immunoglobulin genes and it is estimated that the AID leads to a mutation rate in the variable regions that is approximately 1 million times higher than would occur by spontaneous mutation alone [168]. The process of somatic hypermutation carries two risks to the cell, firstly of causing inappropriate changes to the immunoglobulin gene that lead to ineffective B cell receptor production and these cells are generally removed by apoptosis. The second risk is that the somatic hypermutation activity is not completely restricted to the immunoglobulin variable genes and can cause unwanted and potentially hazardous mutations at other sites, increasing the risk of lympomagenesis [148].

In contrast to the non-chemotherapy curability of B cell malignancies arising in cells that have completed VDJ rearrangement but not effectively proceeded to somatic hypermutation, three B cell malignancies derived from B cells whilst they are undergoing somatic hypermutation all have routine high cure rates with combination chemotherapy.

Genetic analysis indicates that the diagnosis of diffuse large B cell lymphoma DLBCL contains at least 3 major subtypes, germinal centre, activated B cell and primary mediastinal that can be distinguished by their genetic signatures, cell of origin and also by their proximity to the key genetic events in B cell development [169,170]. The impact of the cell of origin of the lymphoma in terms of response and cure rate to first line and second line chemo therapy treatment has been clearly demonstrated, with overall cure rates in germinal centre DLBCL of approximately 80% compared to only 50% in the activated B cell form [170,171]. In the germinal centre DLBCL it is apparent that the somatic hypermutation process has not only commenced but remains active during the natural history of the malignancy as demonstrated with serial biopsies show the evolution of the changes in immunoglobulin gene hypervariable regions [172].

Whilst DLBCL is potentially curable in all age groups, the cure rates for DLBCL decline significantly with increasing patient’s age in a parallel fashion as seen in B-ALL. At the age of 15–49 the reported cure rate is approximately 75%, but falls to 52% for those aged 75 and over [173] In a similar process to that seen in VDJ recombinase activity and B ALL cure rates, the level of activity of SMH system has been demonstrated to fall dramatically in B cells with increasing patient age and this may be linked to the fall in the cure rates of DLBCL seen in older patients [174].

Burkitt’s lymphoma appears to arise in germinal centre B cells that have undergone somatic hypermutation of the VH genes and is characterised by translocation of the c-myc oncogene [175]. There is a difference in the degree of somatic hypermutation and antigenic selection with higher levels seen in the endemic rather than the related sporadic forms [176]. Data indicates that in the sporadic form of Burkitt’s lymphoma the cells are early centroblasts that normally would be proceeding with additional somatic hypermutation but are blocked by the onset of the malignant process. In contrast in the endemic or EBV associated form, the cells have completed somatic hypermutation and starting to mature [176] and the EBV infection and the expression of EBNA-1 acts as an inhibitor of apoptosis [177].

The overall cure rate in Burkitt’s lymphoma with intensive combination chemotherapy such as CODOX-M/IVAC (cyclophosphamide, vincristine, doxorubicin, and high-dose methotrexate, alternating with ifosfamide, etoposide, and cytarabine) with Rituximab is in the region of 77–85% for adults [43].

In Hodgkin’s lymphoma the actual malignant cells make up only a very small proportion of the cell mass and studies on the accurate assessment of the cells of origin and key genetic events and mutations has lagged behind that of non-Hodgkin’s lymphoma. The cells of origin of classical Hodgkin’s disease is confirmed as germinal centre B cells which have completed VDJ rearrangement, with no ongoing recombinase activity [178] and have also undergone somatic hypermutation [179,180]. However it is apparent that these cells are unable to make functional immunoglobulin [179]. Normally in this situation failed germinal centre B cells would be destined to undergo natural apoptosis [181]. However in Hodgkin’s lymphoma these cells with these ‘crippling’ mutations of the immunoglobulin chain are generally viewed as failed germinal centre B Cells that are pre-apoptotic but prevented from progressing to an apoptotic death by EBV infection [181,182] It may be that the loss of the classical B cell phenotype seen in Hodgkin’s disease is a result of the cell moving part of the way down the apoptotic pathways but with the completion of the normal apoptotic pathway blocked as a result of EBV infection [183,184].

The most common regimen for the treatment of Hodgkin’s lymphoma Hodgkin’s is ABVD, which comprises doxorubicin, bleomycin, vinblastine and dacarbazine, which are all old classical chemotherapy drugs [41]. Overall the disease has a chemotherapy cure rate of approximately 80% [185] and it would appear that the addition of the chemotherapy induced DNA damage is sufficient to alter the DNA repair/apoptosis balance to fully effective apoptosis and in doing so overcome the obstacle of the EBV driven anti-apoptotic signaling.

#### Post DNA manipulation B cell malignancies

After the completion of somatic hypermutation and class switching, B cells move towards exiting the germinal centre and move through the lymphoplasmacytic differentiation to become an antibody secreting plasma cell. Each of the malignancies arising after this stage in B cell development, whilst frequently responsive to chemotherapy nearly all cases are not curable with chemotherapy.

The most common of the post VDJ post somatic hypermutation malignancies is follicular lymphoma. Classically the cell of origin for a follicular NHL is considered to be the centrocyte of the germinal centre and involves a cell that has completed somatic hypermutation and class switching and is set on the next stage of development. Clinically follicular lymphoma is characterised by a slow pattern of growth and excellent response to chemotherapy treatment but chemotherapy does not result in cure [186].

In terms of the unique genetic events, follicular lymphoma is demonstrated to carry rearranged immunoglobulin genes that have undergone somatic hypermutation. Whilst in germinal centre DLBCL the process of somatic hypermutation appears to remain ongoing and active in the malignant cells [172,187], in follicular lymphoma the clinical data from repeat analysis of immunoglobulin heavy chain gene sequences through the natural history indicates that whilst AID is still expressed there is little or no on-going somatic hypermutation in follicular lymphoma cells, [188-190]. This divergent finding regarding the ongoing activity of the somatic hypermutation process between diffuse large B cell lymphoma and follicular lymphoma appears to coincide with the differing expectations of the outcome of chemotherapy treatment between these two similar malignancies.

The other post somatic hypermutation B cell malignancies include activated B cell diffuse large B cell lymphoma, MALT lymphoma, lymphplasmacytic lymphoma and myeloma. These malignancies vary significantly in their natural history and response to chemotherapy treatment.

Of these activated B cell DLBCL lymphoma has a significant cure rate with chemotherapy with approximately 40% of patients cured compared to 75% of these with germinal centre DLBCL [170]. In the activated B cell form it is apparent that the somatic hypermutation process is generally completed and the activity of AID has declined significantly but is still present but there is no ongoing somatic hypermutation occurring [172,190]. The role of the apoptosis associated with somatic hypermutation is less well clarified and we could hypothesise that the significant but lower cure rates seen in activated B cell DLBLC is the result of some of the somatic hypermutation associated apoptotic pathways having some appreciable residual activity in a proportion of cases.

Nodular lymphocyte predominant Hodgkin’s lymphoma (NLPHL) is a more recently described diagnosis that makes up approximately 5% of Hodgkin’s disease cases and has a similar morphology to classical Hodgkin’s but differs with a lack of expression of CD15 and CD30 [191]. In NLPHL the immunoglobulin genes have undergone VDJ rearrangement and somatic hypermutation and in contrast to classical Hodgkin’s Lymphoma the L and P cells of NLPHL frequently express surface immunoglobulin [191,192].

The chemotherapy management of NLPHL is usually with ABVD and for patients with advanced disease chemotherapy the overall 10 year disease free survival is approximately 70% which is similar to contemporary results for patients with classical Hodgkin’s lymphoma [193].

In contrast to classical Hodgkin’s lymphoma in which the B cells have completed somatic hypermutation and express little AID activity, in NLPHL there remains significant ongoing AID and SHM activity [194,195]. Whilst classical Hodgkin’s lymphoma appears to be a failed B cell that is prevented from undergoing natural apoptosis by EBV infection, it is likely that the marked chemosensitivity of NHLPL, which characteristically EBV negative [196] stems from the same route as that of DLBCL with ongoing AID activity and the sustained presence of the pro-apoptotic pathways associated with the somatic hypermutation process.

#### MALT and lymphoplasmacytic lymphoma

MALT lymphomas have undergone VDJ rearrangement and completed somatic hypermutation of their immunoglobulin genes [197] and have a relatively indolent natural history. Chemotherapy treatment with the regimens used in follicular lymphoma frequently brings long lived responses but the majority of cases if followed sufficiently long will relapse [198].

Similarly lymphoplasmacytic lymphoma (Waldenstroms macroglobulinaemia) arises from mature B cells that have rearranged VH genes, have undergone somatic hypermutation [199] but have failed to complete class switching [200-202] This malignancy also has a good response rate to chemotherapy but is not chemotherapy curable and has a shorter natural history that follicular NHL or MALT with a median survival of approximately 8 years [203].

#### Hairy cell leukemia

Hairy cell leukemia [HCL] is a rare B cell malignancy that is dramatically responsive to DNA damaging chemotherapy. The modern standard of care is chemotherapy with cladribine and a single cycle of therapy brings a complete response rate of 91% and an average response duration of 98 months [204,205]. However given sufficient follow up, nearly all patients will relapse and require additional therapy. The cell of origin of hairy cell leukemia appears to be a mature B cell that has arrested at the point of isotype switching and individual clones of hairy cell leukemia frequently show staining for more than one isotype of surface immunoglobulin [206]. In HCL there appears to be a degree of ongoing AID expression but there is relatively little clonal variation indicating that the functional SHM process has halted [207].

More recent data has indicated that the BRAF protein that is involved in signal transduction from the B cell receptor has a characteristic V[600]E mutation in nearly all cases of HCL a finding that is not present in any other lymphoid malignancy [208]. This mutation results in constitutive activation of the MEK-ERK pathway that results in a strong survival signal and avoidance of apoptosis [209,210]. As a result it could be postulated that hairy cell leukemia shares a similar scenario to Hodgkin’s lymphoma and ALK+ T cell lymphoma that it is a cell that is blocked from natural apoptosis by the presence of in this case the BRAF V[600]E mutation. The dramatic response to chemotherapy suggests that the DNA damage can shift the balance to highly effective apoptosis, using the mechanisms that are still partially in place linked to the AID activity that is on-going, albeit at a lower level than is present in DLBCL [206,211].

In keeping with other B cell malignancies, hairy cell leukemia has a small subgroup with unmutated immunoglobulin genes. These cases make up less than 5% of the cases of hairy cell leukemia and have a very poor response to chemotherapy treatment and can be refractory to first line cladribine therapy [212].

#### Myeloma

Similarly to the other malignancies of mature B cells, genetic analysis indicates that the malignant cells in myeloma have completed somatic hypermutation and have no ongoing genetic activity [213-215]. The modern treatment of myeloma centres on the use of proteasome inhibitors that has transformed the response rates and median survival but hasn’t changed the incurable nature of the malignancy. Data from and early study with the addition of Bortezomib to cytotoxic chemotherapy indicates this drug has improved complete response rates from 4% to 30% and first line progression free survival 14 months to 18.3 months but that myeloma remains an incurable malignancy [216].

### T cell malignancies

Despite the similar numbers of B cells and T cells, T cell malignancies are relatively rare comprising approximately 10% of the total cases of lymphoma [217]. In contrast to the three processes of VDJ rearrangement, somatic hypermutation and class switching occurring in B cells, T cells have a simpler pattern of genetic activity to achieve their variation in antigenic recognition with only rearrangement of the V and J regions in the TCR alpha chain whilst the TCR beta has the fuller rearrangement of the VDJ components similarly to a B cell [130].

In a parallel fashion to that seen in B cell malignancies the rise in chemotherapy curability coincides with the onset of V[D]J rearrangements in the T cell receptor genes. As discussed previously the rare cases of acute undifferentiated leukemia that arise from the primitive haematopoietic stem cells, that are yet to undergo effective immunoglobulin gene or TCR VDJ rearrangement the chemotherapy cure rates are relatively low. In contrast in acute T cell ALL the V[D]J genes are recombined in nearly 100% of cases [218] and this malignancy has a high cure rate similar to B ALL with 70% for those aged 5–19, 50% aged 20–39 and 20% aged 60 and above [151]. Of interest a recently identified poor prognosis sub group of T ALL has been demonstrated to be T cell receptor negative and is likely to arise prior to the VDJ process becoming fully active [219] and hence has much lower apoptotic potential than T ALL arising during VDJ rearrangement.

Of all the T cell malignancies arising later than T ALL in development, the ALK + ve anaplastic large cell lymphoma is the only one with a significant chemotherapy cure rate. The ALK + ve form of anaplastic large cell lymphoma is usually treated with CHOP chemotherapy and long term survival rates of 70–86% are reported [44,220].

This diagnosis has a similar natural history to classical Hodgkin’s lymphoma. ALK + ve large cell lymphoma arise from T cells that have undergone V[D]J recombination but have failed to produce a functional T cell receptor [221]. Usually these cells would be destined to undergo natural apoptosis in a similar manner to the failed B cells in Hodgkin’s lymphoma [222]. However in the ALK + ve large cell lymphoma the cells have a t[2;5][p23;q35] reciprocal translocation that activates the anaplastic lymphoma kinase gene that leads to abnormal proliferation and resistance to apoptosis [223,224]. As a result the malignant cells are balanced between the pressures and pathways for apoptosis and proliferation and the introduction of radiation or chemotherapy induced DNA damage can successfully tip the balance to apoptosis, cell death and frequently curative treatment.

The rarer form of ALK –ve large cell lymphoma also has clonally rearranged T cell receptor VDJ genes and similarly to ALK + ve large cell lymphoma does not have cell surface T cell receptors [221]. It is unclear why these ALK -ve cells do not undergo natural apoptosis as a result of the failed TCR gene recombination but of interest the 5 year overall survival for ALK –ve T cell lymphoma at 49%, whilst significantly lower than that of ALK + VE [70%] is significantly higher than the 32% reported other peripheral T cell malignancies [225].

There are a wide range of other malignancies arising from mature, post V[D]J] T cells, that correspond to other points on the cells development. The natural history and response to chemotherapy of these malignancies including peripheral T cell lymphoma, angioimmunoblastic lymphoma and adult T cell leukemia/lymphoma varies significantly but they each have a poor prognosis with modest 5 year survival rates and no significant cure rates [226].

### Myeloid malignancies

#### Acute myeloid leukemia [AML]

AML arises from myeloblast cells, that are derived from haematopoietic stem cells via the myeloid stem cells and there are approximately 3.7 cases per 100,000 per year with incidence peaks in young children and older adults [227]. The current standard of treatment employs the classical cytotoxic chemotherapy drugs cytarabine and daunorubicin and recent treatment series indicate an overall cure rate of approximately 40% [40].

Superficially it might appear that AML is an exception to the hypothesis on the importance of the unique genetic events on chemotherapy curability, as myeloid cell development whilst complex, does not require any DNA manipulations. However as previously discussed, the process of VDJ rearrangement that occurs during lymphoid development frequently starts in the common haematopoetic stem cell at a point prior to their differentiation into lymphoid or myeloid stem cells. A number of studies have confirmed that these unique genetic events of VDJ rearrangement that are associated with B cell and T cell development are occurring at significant levels in the cells that develop into AML. In studies of AML, genetic analysis indicates that the immunoglobulin heavy chains genes have undergone VDJ rearrangement in 40–50% of cases and that the expression of the VDJ recombinase associated proteins RAG1 and RAG2 was also detectable in approximately 50% of cases [136,228]. More recent data confirms that VDJ recombination had occurred in the AML myeloblasts of 50% of patients and that there was also limited evidence of somatic hypermutation occurring [229]. In keeping with the observation that VDJ rearrangement is a common event in haematopoietic stem cells destined to proceed towards myeloid differentiation, evidence of significant level of earlier VDJ recombination but not somatic hypermutation are found in monocytes and neutrophils from healthy donors [229]. It is possible that this presence of VDJ activity and the associated pro-apoptotic processes may be linked to the high degree of sensitivity in the production of these cells seen during chemotherapy treatment.

Overall the data is limited on the impact of the completion of these immunoglobulin gene VDJ changes on prognosis and response to chemotherapy and it may be that cells that have completed the IGH VDJ process are less sensitive to induction of apoptosis than those with the malignant process occurring whilst VDJ is still occurring [136,228,230]. However the observation that overall AML has a significantly higher cure rate than acute undifferentiated leukemia suggests that the processes that lead to the high cure rates in B ALL and T ALL may share a common pathway with the VDJ rearrangements pathways seen in all 3 types of cell.

#### Chronic myeloid leukemia [CML]

CML is a malignancy of the haematopoietic stem cells and the BCR/ABL translocation is the source of the malignant phenotype and transformation [231,232]. The incidence is approximately 1–2 cases per 100,000 and the peak incidence is in patients in their 60s.

The malignant cells early in the natural history of CML during the chronic phase, have unrecombined VDJ genes but by the time of entry into lymphoid blast crisis the VDJ regions have become rearranged in the majority of cases. In contrast in myeloid transformation of CML, VDJ rearrangement is rare [137,233].

In studies of chemotherapy treatment of the lymphoid blast crisis, performed prior to the introduction of Imatinib, patients were not cured but had a better response to chemotherapy [median survival 12 months vs 4.7 months] than those with non-lymphoid blasts, with the results suggesting that the processes associated with VDJ improve the response to chemotherapy [234].

In contrast to other malignancies, including B ALL and T ALL and AML, which arise in cells around the time of VDJ rearrangement, CML in lymphoid blast crisis is not chemotherapy curable. This divergent outcome compared to the acute leukemias may reflect two key differing elements of the CML lymphoid blast cells. The first significant difference between CML and the acute leukemias is the timing of the VDJ rearrangement and the onset of the malignant phenotype. In the acute leukemias the malignant transformation occurs early in the cells development and concurrently with the VDJ rearrangement and the cells retains the apoptotic sensitivity of these cells. In contrast in CML the VDJ rearrangement occurs much later in the natural history of the malignancy, often many years after the establishment of the malignant phenotype by the BCR/ABL translocation.

The other key difference is the role of the BCR/ABL translocation on inhibiting apoptosis. The major impact of this protein on inhibiting apoptosis is seen in studies where BCR/ABL has been transfected into chemotherapy sensitive cells lines leading to high levels drug resistance [235]. As a result the presence of the BCR/ABL activity may be sufficient to override the apoptotic sensitivity normally seen with active VDJ rearrangement.

#### Other myeloid malignancies

Other myeloid malignancies, including myelodysplastic syndrome, atypical CML, and chronic myelomonocytic leukemia are characterised by poor response to chemotherapy. Analysis of the immunoglobulin and T cell receptor genes in myelofibrosis indicates that the vast majority of cases have unrearranged germ line genes [236,237]. Similarly in acute myelomonocytic leukemia and acute monoblastic leukemia immunoglobulin gene rearrangements are not detected [238] and these malignancies have relatively poor responses to chemotherapy and low cure rates [239].

## Summary and therapeutic implications

The historical explanations for chemotherapy sensitivity and resistance usually include the concepts of rates of growth, mathematical risks of the development of resistance, drug efflux pumps and changes in DNA repair mechanisms. For many oncologists these hypotheses appear to be potentially at odds with the clinical data, which sees patients with a limited range of malignancies routinely cured but other fast growing malignancies including small cell lung cancer and epithelial ovarian cancer responding dramatically to chemotherapy, often achieving a complete remission but uniformly relapsing.

In this review we have examined an additional key factor that appears to impact greatly on the sensitivity and curability of malignancies with chemotherapy treatment; the relationship of the cell of origin of the malignancy to the unique genetic processes of nuclear fusion, gastrulation, meiosis, VDJ rearrangement and somatic hypermutation. Each of the disseminated malignancies that are currently routinely curable with cytotoxic chemotherapy arises from cells with naturally occurring heightened apoptotic potential linked to the unique genetic events, whilst all other metastatic malignancies that arise from cells that are not involved in these processes are not chemotherapy curable.

Whilst it is clear that the historical explanations for chemotherapy resistance retain an important role in the degree of sensitivity and the resistance of some cases of the chemotherapy curable diagnoses to treatment, we would hypothesis that what determines the overall degree of sensitivity is the relationship with these unique genetic events.

The clinical applications of technology to overcome chemotherapy resistance, whether by effecting drug efflux pumps, or replacing mutated apoptotic pathways proteins, such as p53 have shown only modest activity and are these do not form part of routine clinical care [240].

There has been enormous endeavour to spread the dramatic benefits from chemotherapy from these selected chemotherapy curable malignancies to the wider range of cancers by overcoming chemotherapy resistance. Despite this work, there has been no increase in the range of chemotherapy curable malignancies, little routine clinical application of these approaches and now the majority of new oncology drugs are molecularly targeted therapies rather than additional new or improved classical cytotoxic agents.

This raises the issue of whether the development and range of activity of classical cytotoxic drugs is now nearly complete or if new accompanying technologies can allow the mode of action and range of effective targets to be significantly improved.

In terms of extending chemotherapy curability to other malignancies with classical cytotoxic chemotherapy drugs the observation that no more effective therapies have been introduced since 1982 would argue that the development of this technology is near complete. However if the interpretation of the information in this paper is correct, there are within the majority of cancer cells the genes present that could turn the current modest benefits from chemotherapy drug treatment potentially into something much more dramatic, however the technology to achieve this is challenging.

One could hypothesis that if the pro-apoptotic pathways associated with these unique genetic event could be activated in other types of cancer cells then the benefits from chemotherapy could be dramatically improved. Whilst it is difficult to envisage employing the processes supporting nuclear fusion and gastrulation, the processes supporting meiosis and VDJ rearrangement may be more realistically accessible.

It has previously been noted that radiotherapy treatment to fibroblast and lymphoma cells can result in the expression of meiosis related proteins and that the combination of DNA damage and meiosis related proteins can only be tolerated by cells with mutated p53 pathways. In cells with intact p53 the combination of DNA damage and meiosis leads to apoptosis [241,242]. Similarly more recent data indicates that meiotic proteins and meiotic depolypoidization division can occur after radiation induced DNA damage in a wider range of tumour cell lines [243].

Whilst inducing any of these unique genetic events as a therapeutic approach might appear fanciful, recent data shows that a number of non-B cell malignant cells actually express their recombined immunoglobulin genes and can make antibodies, which in some cases have undergone a tumor type repetitive pattern of VDJ rearrangement pattern [244-246]. Additionally it appears that some cells can undergo a limited and atypical form of somatic hypermutation and that measurable levels of AID is still expressed but that ongoing mutation does not appear to be occurring in a number of studied breast cancer cell lines [247]. It is unclear if cancer cells that are making immunoglobulins have any increase in their sensitivity to chemotherapy and treatment outcomes. These findings that the immunoglobulin gene system is at least active in part in a number of tumor types raises the possibility that the fuller activity with the induction of effective apoptosis could be a novel therapeutic strategy.

The effective treatment and cure of the rare chemotherapy curable malignancies has been one of the great achievements of modern medicine, it is possible that with a greater understanding of the processes that make these diagnoses so sensitive to chemotherapy that the benefits could be extended to a much wider group of malignancies by the exploitation of the natural pathways that should be present but not activated in most types of malignant cells.
